# Inflammation-Related Carcinogenesis and Prevention in Esophageal Adenocarcinoma Using Rat Duodenoesophageal Reflux Models

**DOI:** 10.3390/cancers3033206

**Published:** 2011-08-10

**Authors:** Takashi Fujimura, Katsunobu Oyama, Shozo Sasaki, Koji Nishijima, Tomoharu Miyashita, Tetsuo Ohta, Miwa Koichi, Hattori Takanori

**Affiliations:** 1 Gastroenterologic Surgery, Kanazawa University Hospital, Kanazawa, Japan, 13-1 Takaramachi, Kanazawa, Ishikawa 920-8641, Japan; E-Mails: oya-ma@staff.kanazawa-u.ac.jp (K.O.); syozossjp@yahoo.co.jp (S.S.); kojinishijima@yahoo.co.jp (K.N.); tomoharumiya@gmail.com (T.M.); ohtat@staff.kanazawa-u.ac.jp (T.O.); 2 Houju Memorial Hospital, Nomi, Japan, 11-71 Midorigaoka, Nomi, Ishikawa 923-1226, Japan; E-Mail: kmiwa@med.kanazawa-u.ac.jp; 3 Division of Molecular and Diagnostic Pathology, Shiga University of Medical Science, Otsu, Japan, Seta Tsukinowa-cho, Otsu, Shiga 520-2192, Japan; E-Mail: hattori@belle.shiga-med.ac.jp

**Keywords:** inflammation-metaplasia-adenocarcinoma sequence, Barrett's esophagus, duodenogastroesophageal reflux, chemoprevention for esophageal adenocarcinoma, antireflux surgery, proton pump inhibitors, nonsteroidal anti-inflammatory drugs, selective cyclooxygenase-2 inhibitors, thioproline

## Abstract

Development from chronic inflammation to Barrett's adenocarcinoma is known as one of the inflammation-related carcinogenesis routes. Gastroesophageal reflux disease induces regurgitant esophagitis, and esophageal mucosa is usually regenerated by squamous epithelium, but sometimes and somewhere replaced with metaplastic columnar epithelium. Specialized columnar epithelium, so-called Barrett's epithelium (BE), is a risk factor for dysplasia and adenocarcinoma in esophagus. Several experiments using rodent model inducing duodenogastroesophageal reflux or duodenoesophageal reflux revealed that columnar epithelium, first emerging at the proliferative zone, progresses to dysplasia and finally adenocarcinoma, and exogenous carcinogen is not necessary for cancer development. It is demonstrated that duodenal juice rather than gastric juice is essential to develop esophageal adenocarcinoma in not only rodent experiments, but also clinical studies. Antireflux surgery and chemoprevention by proton pump inhibitors, nonsteroidal anti-inflammatory drugs, selective cyclooxygenase-2 inhibitors, green tea, retinoic acid and thioproline showed preventive effects on the development of Barrett's adenocarcinoma in rodent models, but it remains controversial whether antireflux surgery could regress BE and prevent esophageal cancer in clinical observation. The Chemoprevention for Barrett's Esophagus Trial (CBET), a phase IIb, multicenter, randomized, double-masked study using celecoxib in patients with Barrett's dysplasia failed to prove to prevent progression of dysplasia to cancer. The AspECT (Aspirin Esomeprazole Chemoprevention Trial), a large multicenter phase III randomized trial to evaluate the effects of esomeprazole and/or aspirin on the rate of progression to high-grade dysplasia or adenocarcinoma in patients with BE is now ongoing.

## 1. Introduction

A rapid increase in the incidence of esophageal adenocarcinoma (EAC) has become a clinical problem among people of Western countries, especially, Caucasian males [1]. The sequence of events progressing from gastroesophageal reflux disease (GERD) to EAC is thought to involve the development of inflammation-stimulated hyperplasia and metaplasia such as Barrett's esophagus (BE), followed by multifocal dysplasia and adenocarcinoma. These diseases are associated with duodenal juice as well as gastric juice. GERD incidence has risen dramatically, not only in Western countries but also Eastern countries, including Japan, due to the prevalence of obesity, westernized diets, and hiatal hernia. Thus it can be anticipated that these diseases will develop into critical problems in health care systems worldwide.

We have established rodent duodenoesophageal reflux models to develop EAC without using exogenous carcinogens [2,3]. In these models refluxate, including duodenal or duodenogastric juice, first induced strong inflammation, followed by metaplasia and dysplasia, and finally developed adnocarcinoma. Histopathological changes in the inflammation-metaplasia-adenocarcinoma sequence are described and prevention by surgical intervention and chemoprevention are discussed in this paper.

## 2. Esophageal Carcinogenesis Due to Exogenous Carcinogens

The experiments with respect to esophageal carcinogenesis were traditionally carried out using mutagenic nitrosamines such as 2,6-dimethylnitrosomorpholine (DMNM), methyl-n-amylnitrosamine (MNAN), and *N*-nitrosomethylbenzylamine (NMBA). Esophageal carcinoma developed by these carcinogens was histologically squamous cell carcinoma, while it was reported that additional esophagojejunostomy to carcinogens induced adenocarcinoma in distal esophagus [4]. In 1992, Attwood *et al.* investigated whether gastroesophageal or duodenoesophageal reflux influences the prevalence and differentiation of induced esophageal cancer in nitrosamine-treated rats [5]. They reported that the rate of squamous carcinoma was 25–30% for rats with either DMNM or MNAN alone, and 20% for rats with induced gastroesophageal reflux plus DMNM, while the rate of malignant change rose up to 67–80% in rats with induced duodenoesophageal reflux plus either nitrosamine. With duodenoesophageal reflux, 50% of tumors were adenocarcinoma, in contrast to 100% squamous differentiation of tumors in rats given the carcinogens with esophagogastroplasty, which was supposed to induce gastric reflux alone, or no operation. These results indicated that the duodenoesophageal reflux increased the frequency and changed the histologic type of esophageal cancer in nitrosamine-treated rats, suggesting that duodenal refluxate plays a role as a co-carcinogenic factor in the development of esophageal adenocarcinoma.

## 3. Esophageal Adenocarcinoma induced by Duodenoesophageal Reflux Alone

**Figure 1.**
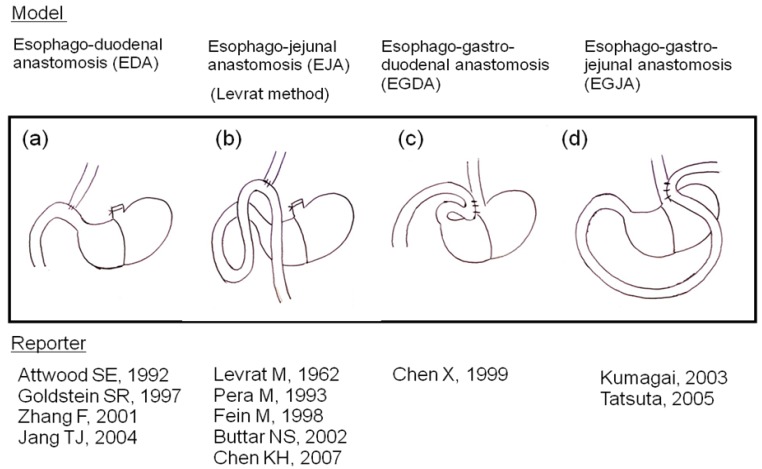
Rodent models of duodenogastroesophageal reflux. (**a**)–(**d**) Several kinds of duodenogastroesophageal reflux were established using rodent models. Duodenum or jejunum is anastomosed with blind end of esophagus or esophagogastric junction. These four models are characterized by combined refluxate including not only duodenal juice but also gastric juice.

We were the first to report development of columnar epithelial metaplasia and mucinous adenocarcinoma, as well as squamous cell carcinoma, using a rodent duodeno-forestomach or duodeno-glandular-forestomach reflux model to put duodenal juice into the esophagus without exogenous carcinogens [2]. Several researchers have reported many kinds of reflux models (Figure 1) and agreed with our idea that carcinogen is unnecessary for esophageal carcinogenesis in rodent reflux models [6,7].

**Figure 2.**
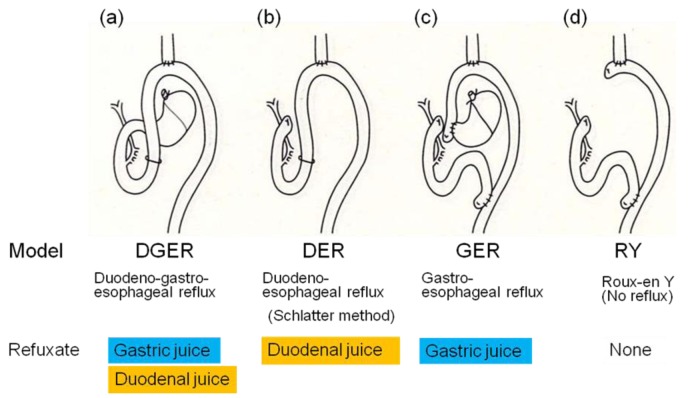
(**a**) Duodenogastroesophageal reflux model (DGER) has regurgitation of both duodenal and gastric juices; (**b**) Duodenoesophageal reflux model (DER) has regurgitation of duodenal juice alone; (**c**) Gastroesophageal reflux model (GER) has regurgitation of gastric juice alone; (**d**) Roux-en Y esophagojejunostomy model (RY) does not have either regurgitation.

Subsequently, we have established a rodent duodenoesophageal reflux model to produce BE and EAC without administration of any carcinogens and investigated the incidence of esophageal adenocarcinoma in four types of rodent models, shown in Figure 2, to elucidate which component is responsible for development of EAC, duodenal juice or gastric juice [3]. The duodenoesophageal reflux model (DER) has regurgitation of duodenal juice alone, while the gastroesophageal reflux model (GER) has regurgitation of gastric juice alone. The duodenogastroesophageal reflux model (DGER) has regurgitation of both duodenal and gastric juices, but neither reflux occurs in the Roux-en Y esophagojejunostomy model (RY).

**Figure 3.**
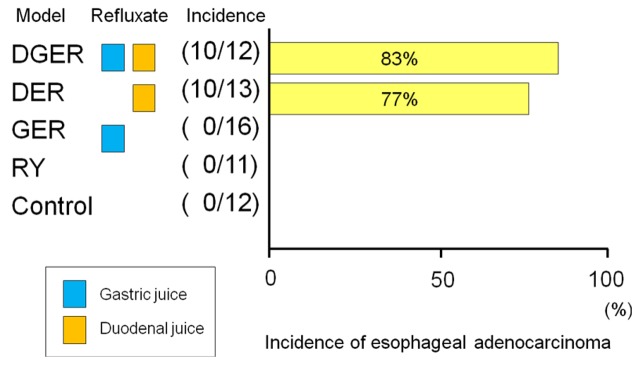
The incidence of esophageal adenocarcinoma in the DGER or the DGR was significantly higher than in the GER or the RY (p < 0.001). Esophageal adenocarcinoma developed only in models with refluxate including duodenal juice. No exogenous carcinogen was administered in this experiment.

The incidences of esophageal adenocarcinoma in the DER, GER, DGER, and RY model were 7/13 (54%), 0/16 (0%), 9/12 (75%), and 0/11 (0%), respectively (Figure 3). Adenocarcinoma developed only in models with refluxate including duodenal juice. Fein *et al.* also reported that 48% of animals receiving esophagojejunostomy developed esophageal adenocarcinoma at the anastomotic site without carcinogen administration [6]. Cancer prevalence tended to be lower in animals receiving acidified water (pH 1.8), suggesting that gastric juice has a negative effect on the carcinogenesis of EAC. But protective effect of gastric juice on esophageal adenocarcinogenesis might depend upon experimental designs such as a use of carcinogen, experimental term, and rat strain [8]. Taken together, we can conclude that exogenous carcinogens are not necessary for cancer development and duodenal juice rather than gastric juice is essential to develop EAC in rodent models.

## 4. Inflammation-Metaplasia-Adenocarcinoma Sequence

**Figure 4.**
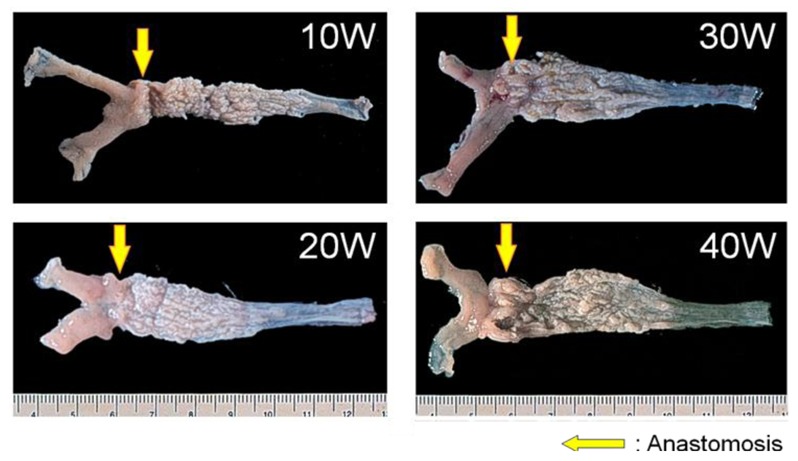
Coarse and tubercular lesion develops near the anastomosis at the 10th week after surgery and extends to the proximal esophagus in the rat duodenoesophageal reflux model. A large protruding lesion appears near the anastomosis at the 40th week after surgery.

**Figure 5.**
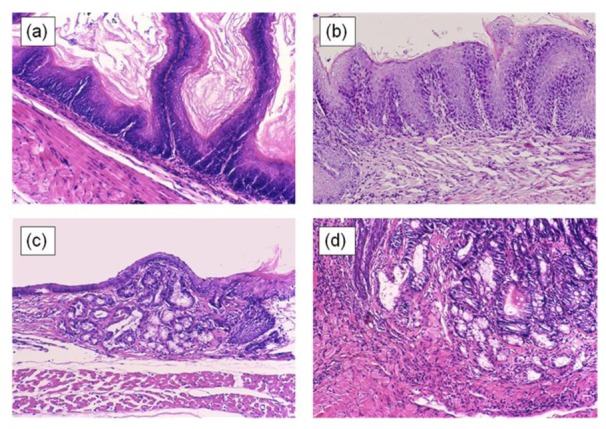
Microscopic findings in the rat duodenoesophageal reflux model. (**a**) Regenerative thickening, esophageal epithelium with more than double the normal thickness, together with acanthosis, abnormal extension of papillae toward the mucosal surface, and parakeratosis; (**b**) Basal cell hyperplasia, thickening of the basal layer of the squamous epithelium resulting in its occupation of more than 15% of the epithelial layer. The stratified structure of the squamous epithelium is preserved; (**c**) Barrett's esophagus, replacement of squamous epithelium with columnar-lined epithelium consisting of gastric and/or intestinal cells; (**d**) Adenocarcinoma, epithelial growth with cellular and structural atypism, invading into the submucosal layer.

We reported chronological changes of esophageal mucosa (shown in Figures 4 and 5) using the DER model (Figure 2b) [9]. An inflammatory response consisting of infiltration of small round cells, mainly lymphocytes, occurs in the 10th week after surgery and the response is severest around the 20th week. Most of the squamous epithelium shows regenerative thickening from the middle to lower esophagus in the 10th week after surgery, followed by basal cell hyperplasia from the 20th week. These hyperplastic changes accompany mucosal erosions.

In the 20th week, glandular structures are observed scattered throughout the basal layer of the esophageal epithelium in all animals. These glandular structures are comprised of columnar-lined epithelium, which are seen under the proliferative zone and are not rich in mucins. These lesions include cells with GOS-positive, Con A-negative, and HID-AB-negative cytoplasm, similar to the gastric foveolar epithelium.

**Figure 6.**
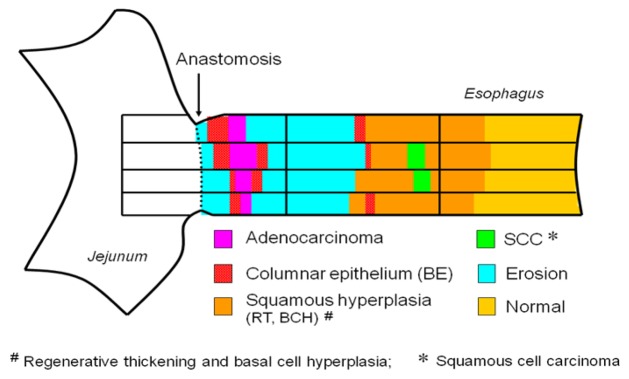
Columnar-lined metaplasia appears near esophagojejunostomy, surrounded by erosion. Adenocarcinoma scatteringly develops in the area showing columnar-lined epithelium. On the other hand, squamous cell carcinoma develops in the area showing squamous hyperplasia, which is located proximal to the columnar-lined metaplasia.

In the 30th week, basal cell hyperplasia and erosion are admixed within a region from the anastomosis toward the oral side. In some of these squamous epithelia, the gland ducts advance deeply within the mucosa and form cystic proliferation. Metaplasia of gastric pyloric glands are observed, in which the cells at the leading region showed increased mucin levels and became HID-AB-positive. The gland tissues also develop to the luminal side of the digestive tract and occupy the whole esophageal mucosal layer, replacing squamous epithelium. Goblet cells positive for HID-AB, as well as GOS and Con A, emerge in complete columnar-lined metaplasia, corresponding to the specialized columnar epithelium of BE in human, suggesting intestinal metaplasia. These specialized columnar epithelia are seen from the 30th week and develop in all animals by the 40th week (Figure 6).

Adenocarcinoma is observed near the esophagojejunal anastomosis from the 40th week. It is surrounded by specialized columnar epithelium. Squamous cell carcinoma and adenosquamous carcinoma also develop from the 40th week, but compared to EAC, these lesions always accompany basal cell hyperplasia and arise proximal to the area of columnar-lined epithelium.

**Figure 7.**
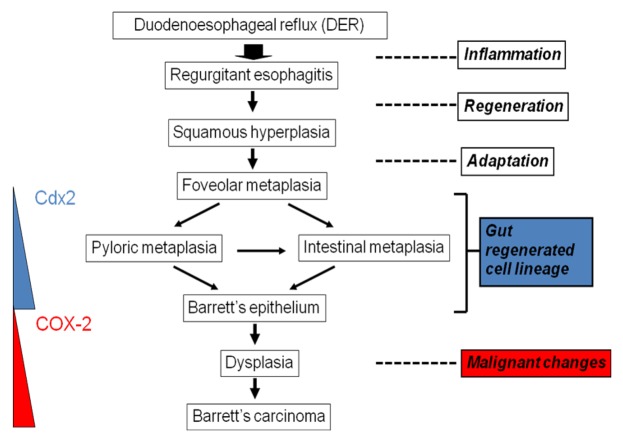
Metaplasia-dysplasia-carcinoma sequence in reflux model. Duodenoesophageal or duodenogastroesophageal reflux induces strong inflammation. Esophageal epithelium is usually regenerated with squamous cell, but sometimes and somewhere replaced with metaplasia, such as foveolar, pyloric and intestinal metaplasias. The Cdx2 is supposed to drive this adaptive response. Furthermore, specialized columnar epithelium, so-called Barrett's esophagus, progresses to mild dysplasia, severe dysplasia, and carcinoma. Many factors including COX-2, and nitric oxide stimulate these malignant changes.

This inflammation-induced metaplasia in the rat reflux model is characterized by pyloric-foveolar metaplasia followed by specialized columnar epithelium. This morphological change is a common phenomenon and may be viewed as a regenerative response to the gastrointestinal mucosal injury. The concept of this pathological sequence is named ‘gut regenerative cell lineage (GRCL)’ (Figure 7) [10]. In general, metaplasia appears to be an adaptive response to toxic agents induced by chronic inflammation because the metaplastic tissue is more resistant than the original tissue. Eberhard *et al.* described that metaplasia can result from the abnormal differentiation of stem cells or from transdifferentiation of mature cell type into another type of mature cell. [11] The GRCL may be one of such metaplasias in gastrointestinal tract. Recently, it was reported that Cdx2 was expressed not only in BE, but also in several pyloric gland and foveolar metaplastic cells [12]. Cdx2 is one of the homeobox genes related with intestinal development, differentiation, and maintenance of intetinal phenotype. Not only bile acids such as deoxycholic acid and cholic acid, but also acid activate Cdx2 promotor in several cell lines [13,14]. Taken together, Cdx2 gene might drive intestinalization of squamous cell epithelium by the GRCL.

Souza *et al.* summarize the story of inflammation-induced metaplasia from the viewpoint of acid-bile reflux and specific gene expressions [15]. Acid-peptic damage to the tight junctions between esophageal squamous epithelial cells results increased epithelial permeability. Acid, bile salts, and inflammatory mediators could be easily infiltrated into the deep layer of esophageal mucosa. This exposure might trigger the expression of Cdx1, Cdx2, and other genes related with intestinalization in stem cells, while expressions of BMP-4 and other morphogenic factors increase in stroma cells. It is suggested that the interaction between Cdx genes and BMP-4 mediates the development of Barrett's metaplasia. On the other hand, CpG island hypermethylations in various genes rather than gene mutations involve the development of BE into EAC. Barrett's adenocarcinoma indicates higher incidences of hypermethylation in the E-cadherin, human MutL homolog 1, p16/INK4A and O^6^-methylguanine-DNA methyltransferase than BE [16].

## 5. Prevention of Barrett Esophagus and Esophageal Adenocarcinoma

### 5.1. Surgical Intervention

**Figure 8.**
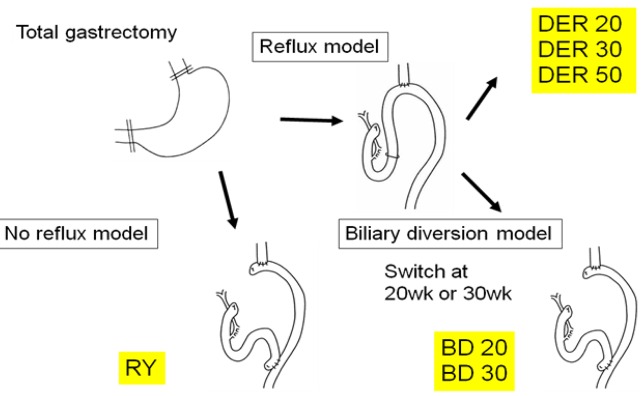
All animals first received total gastrectomy. (1) The DER 20, DER 30, or DER 50 group was reconstructed by esophagojejunostomy to induce the DER (Figure 2b), followed by killing after 20, 30, or 50 weeks, respectively; (2) biliary diversion (BD) procedure, converted from the same DER operation (Figure 2b) to the RY method (Figure 2d), to avoid bile regurgitation into the esophagus at week 20th (BD 20) or week 30th (BD 30), followed by euthanasia at 50 weeks after initial operation; or (3) The RY group was reconstructed by RY method (Figure 2**d**), followed by killing after 50 weeks served as controls.

It remains controversial whether or not antireflux surgery could induce the regression of BE and prevention of esophageal cancer. We performed surgical conversion from reflux model to antireflux model to examine whether elimination of duodenal reflux could regress BE and/or prevent the development of EAC (Figure 8) [17].

Two hundred F344 male rats underwent one of following three operations: (1) total gastrectomy and esophagojejunostomy to induce DER (Figure 2b), followed by killing after 20, 30, or 50 weeks; (2) biliary diversion (BD) procedure, converted from the same operation to RY method (Figure 2d), to avoid bile regurgitation into the esophagus at week 20th or week 30th, followed by euthanasia at 50 weeks after initial operation; or (3) total gastrectomy reconstructed by RY (Figure 2d), followed by killing after 50 weeks served as controls.

**Figure 9.**
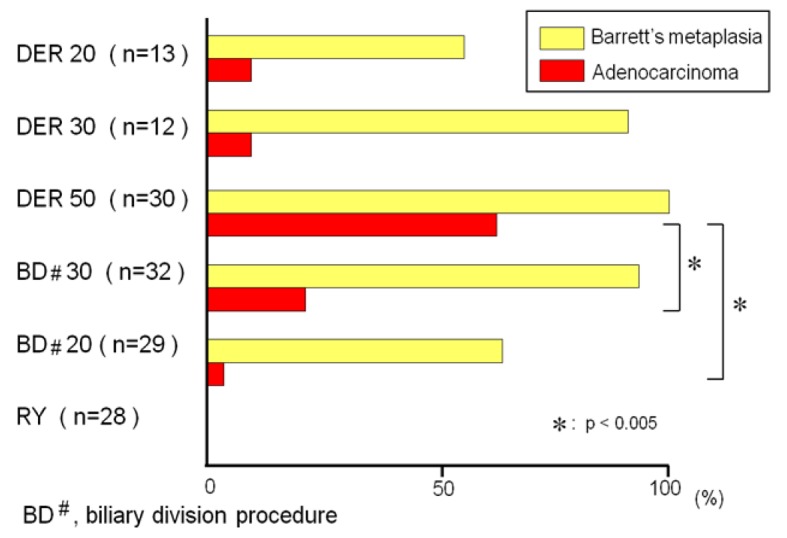
There was no difference in the incidence of Barrett's metaplasia between the animals sacrificed at week 20th (54%) or 30th (92%) in the DER model and the animals undergoing biliary diversion procedure at week 20th (62%) or 30th (94%). But the incidence of adenocarcinoma was significantly lower in the rats that received the biliary diversion procedure after 30 (19%) and 20 weeks (3%) than in the rats that had DER for 50 weeks (60%) (p < 0.005).

The animals exposed to DER for 20, 30, and 50 weeks developed BE in 54%, 92%, and 100%, respectively. The incidences of BE in the animals that underwent biliary diversion at week 20th and 30th were 62% and 94%, respectively. There was no difference in the incidence of BE between the animals sacrificed during the 20th or 30th week in the DER model and the animals undergoing biliary diversion procedure at week 20th or 30th. But the incidence of adenocarcinoma was significantly lower in the rats that received the biliary diversion procedure than in the rats that had DER for 50 weeks (Figure 9). These results suggested that the conversion procedure from reflux model to antireflux model does not lead to regression of BE, but prevents the development of EAC in the rats.

In a clinical study Hofstetter *et al.* investigated the long-term outcome of antireflux surgery in patients with BE [18]. Seventy-three of 85 patients underwent Nissen fundoplication and were assessed at a median follow-up of 59 months. They reported that dysplasia had regressed in nearly half of the patients in whom it was present before surgery, intestinal metaplasia disappeared in 14% of patients, and high-grade dysplasia and adenocarcinoma were prevented in all. This result is concordant with our experimental outcomes with respect to prevention of adenocarcinoma. On the other hands, Csendes questioned the preventive effect of antireflux surgery on BE by showing that low-grade dysplasia and adenocarcinoma postoperatively appeared in 6.0% and in 3.4%, respectively, by reviewing 25 articles published from 1980 to 2003 dealing specifically with the surgical treatment of BE [19].

Antireflux surgery for esophageal regurgitation is usually carried out by wrap fundoplication procedures including Nissen-, Toupet-, and Dor-fundoplication. But these procedures are insufficient to block reflux into esophagus because they just make the esophagogastric junction tighter. Therefore a new antireflux operation was invented, which consists of vagotomy, antrectomy (antiacid procedures), and RY gastrointestinal reconstruction (a biliary diversion procedure) [20]. This operation induced a regression of BE from intestinal to cardiac or fundic mucosa in about 60% of patients with no patient progressing to high-grade dysplasia or adenocarcinoma. These results suggest that this operation may become an attractive option as a definitive surgical treatment.

### 5.2. Chemoprevention

Sporn *et al.* coined the term ‘chemoprevention’ in 1976, defining it as arrest or reversal of premalignant cells by using physiological pathways [21]. Recently, some chemical agents such as proton pump inhibitors (PPIs), nonsteroidal anti-inflammatory drugs (NSAIDs), selective cyclooxygenase-2 inhibitors (COXIBs), green tea, retinoic acid and thioproline have been reported to prevent EAC [22]. We performed several experiments by our rodent model using rabeprazole, thioproline or celecoxib to prevent inflammation-metaplasia-adenocarcinoma (IMA) sequence.

#### 5.2.1. Proton Pump Inhibitors (PPIs)

A probable alternative to antireflux surgery is acid suppression by anti-acid medicines such as histamine H2-receptor antagonists and PPIs. Miyashita *et al.* evaluated the chemopreventive effects of rabeprazole (RBZ) on esophageal carcinogenesis using a rat duodenogastroesophageal reflux model (Figure 2a) [23]. The incidences of Barrett's metaplasia and esophageal cancer in the control group were 100% (23/23) and 74% (17/23), respectively, significantly higher than 65% (11/17) and 29% (5/17) in the RBZ group. These data supported that RBZ could prevent not only BE but also esophageal cancer.

Epidemiologic studies showed that long-term use of PPI was associated with lower rates of dysplasia and adenocarcinoma in patients with Barrett's esophagus [24,25]. On the other hand, two case-control studies from the United Kingdom and the United States failed to prove decreases in the risk of EAC by acid suppression [26,27]. Furthermore, a large-scale review to compare antireflux surgery with medical therapy was carried out to disclose the proportion of patients developing progression or regression of BE and/or dysplasia as well as the incidence of adenocarcinoma. There was no difference between two therapies in the incidence of EAC, while the probability of progression in surgical patients was low compared with medical patients (2.9% *vs.* 6.8%) and the probability of regression in surgical patients was high compared with medical patients (15.4% *vs.* 1.9%) in medical patients. Thus it remains to be elucidated whether anti-acid medication is really effective to suppress the development of dysplasia and adenocarcinoma.

The AspECT (Aspirin Esomeprazole Chemoprevention Trial), a large multicenter phase III randomized trial, to evaluate the effects of esomeprazole and/or aspirin on the rate of progression to high-grade dysplasia or adenocarcinoma in patients with BE is now ongoing [28]. The combination of a PPI and a NSAID seems very attractive because both drugs are strongly expected to show chemopreventive effects on esophageal carcinogenesis.

#### 5.2.2. Thioproline

Thioproline (TPRO) is one of the nitrite-trapping agents which reduce the production of carcinogenic *N*-nitroso-compounds. Nitrate-reducing bacteria, which convert dietary nitrate to nitrite, overgrow in the stomach when intragastric acidity is decreased by atrophic gastritis or gastrectomy. Nitrite combines with amines and amides in foods to produce *N*-nitroso-compounds, while TPRO could inhibit this reaction competitively.

We examined whether TPRO inhibits the inflammation-metaplasia-adenocarcinoma sequence or not [29]. Fisher 344 rats receiving esophagojejunostomy (Figure 2a) preserving whole stomach were fed with a thioproline-containing diet (the TPRO group) or control diet (the control group) for 45 weeks. Incidences of BE and EAC in the TPRO group were 67% and 17%, while they were 94% and 69% in the control group. A significant reduction in the incidence of EAC was confirmed, but there was no difference in the incidence of BE between two groups. These results suggested that TPRO could prevent only development from BE to adenocarcinoma, but could not suppress inflammation or BE. Kumagai *et al.* reported a similar result using another rat model (Figure 1d) [30]. They showed that the incidence of EAC in the control group was 38.9% compared to no adenocarcinoma in the TPRO group and iNOS protein was overexpressed in BE of both groups, speculating that TPRO inhibited the production of not only nitroso-compounds by nitrate-reducing bacteria but also reactive nitrogen species, such as NO, ONOO^-^ and *N*-nitroso-compounds, derived from duodenal refluxate. NO is produced from nitrite by iNOS and induces chronic inflammation. Recently it was reported that NO is related with BE or EAC by mucosal injury at the esophagogastric junction [31].

#### 5.2.3. NSAIDs, Especially COX-2 Inhibitors (COXIBs)

It is reported that the risk of EAC is reduced in users of aspirin and other nonsteroidal anti-inflammatory drugs (NSAIDs) [32]. Multiple lines of evidence have demonstrated that overexpression of COX-2 is observed in EAC as well as squamous cell carcinoma [33,34]. COX-2 expression is gradually up-regulated with development of esophageal lesions, from 75% in metaplasia, to 83% in low-grade dysplasia and to 100% in high-grade dysplasia and EAC [35]. Duodenogastric reflux to esophagus contributes to the development of these diseases [36] and BE patients have higher bile acid levels in the stomach than healthy controls and GERD patients without BE [37]. These observations strongly indicate that duodenal juice including bile is associated with the inflammation-metaplasia-adenocarcinoma sequence. In particular, bile acid is likely to play a pivotal role. Zhang *et al.* reported that bile acids induced COX-2 mRNA, followed by COX-2 protein and PGE_2_ production [38]. More recently, it was demonstrated that unconjugated bile acids such as chenodeoxycholic acid and deoxycholic acid induced CREB and AP-1 dependant COX-2 expression in BE and EAC through PI3K/AKT and ERK 1/2 pathway [39].

To investigate COX-2 involvement in carcinogenesis by duodenoesophageal reflux three experiments using a rodent reflux model with NSAIDs or COXIBs were reported (Table 1). Sulindac, one of NSAIDs, is reported to have a chemopreventive effect against the formation of EAC in the rat EGDA model (Figure 2c) possibly through its inhibition of COX in 2002 [40].

**Figure 10.**
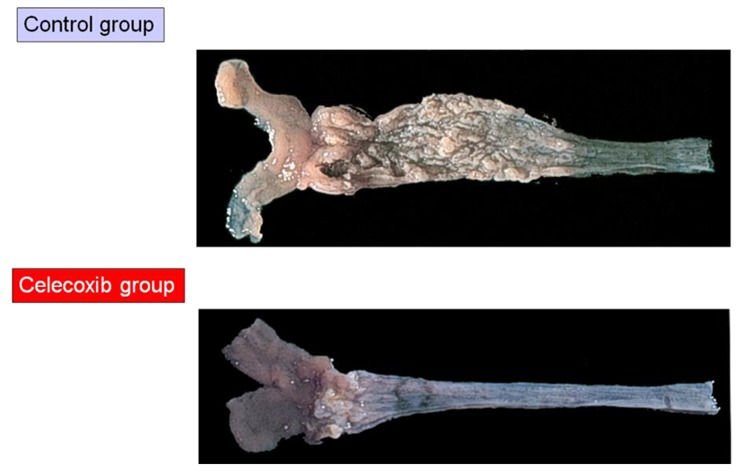
The figure showed representative photographs of macroscopic appearance in the control and celecoxib groups at the 40th week. The esophageal mucosa in the control group is obviously coarse and thick compared with that in the celecoxib group.

Buttar *et al.* first showed a preventive effect on EAC by a COXIB, MF-tricyclic, in a rat model of BE and EAC by duodenogastroesophageal reflux (Figure 1b) [41]. In their report, MF-tricyclic prevented the development of EAC, but did not suppress the prevalence of BE. On the other hand, we used another COXIB, celecoxib (CLX), which is known to be already prescribed for patients with familial adenoma polyposis to prevent colorectal polyps [42]. Rats receiving esophagojejunostomy after total gastrectomy (Figure 2b) were fed with CLX-contained diet (CLX group) or control diet (Control group) for 40 weeks (Figure 10). Incidences of BE and EAC in the CLX group were 25% and 0% while these were 89% and 47% in the control group (Table 2).

**Figure 11.**
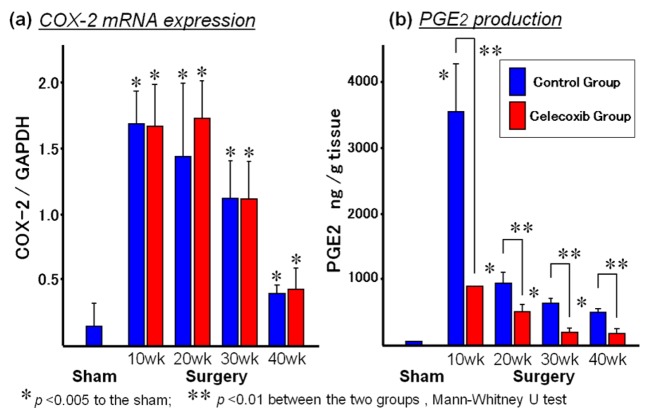
(**a**) COX-2 mRNA was overexpressed during whole period of the experiment compared to sham operation. In particular a surge of COX-2 mRNA emerged during the 10th to 20th week. It seems that a very strong inflammation occurred in the early phase in inflammation-metaplasia-carcinogenesis sequence. Celecoxib did not suppress COX-2 mRNA at all; (**b**) On the other hand, PGE_2_ product markedly increased throughout the experiment. Celecoxib dramatically suppressed the PGE_2_ level.

Thus we demonstrated that CLX suppressed not only the development of EAC, but also that of reflux esophagitis, and BE by suppressing PGE_2_ production in rodent model (Figure 11). Our data indicates that CLX could postpone inflammation-metaplasia-adenocarcinoma sequence itself.

It remains unclear why there is a difference in the result between two experiments because they differ in rat strain, operative procedure, chemical agent used and so on. (Table 1) Three hypotheses accounting for reduced incidence of esophageal cancer by COX-2 inhibitor are suggested, that is, to inhibit the development of BE from reflux esophagitis, to inhibit the development of dysplasia in BE, or to inhibit the process of carcinogenesis in dysplasia. Buttar *et al.* speculated that MF-tricyclic did not affect the development of BE because of no difference in the incidence of BE between the MF-tricyclic group and the control group in their experiment, but they also suggested a tumor overgrowing and replacing an area previously occupied by dysplasia might have falsely lowered the rate of dysplasia in the control group. Recently it was reported that continuous CLX administration significantly reduced not only COX-2 protein expression, but also Cdx2 and MUC2 protein immunoreactivity, and furthermore decreased the extent of intestinal metaplasia of stomach using an *H. pylori*-infected gerbil model [43]. These results strongly supported that CLX could postpone IMA sequence itself by suppressing intestinalization by Cdx2 activation.

These data stimulated a clinical chemoprevention study for the patients with BE. Kaur *et al.* reported that administration of 25-mg/day rofecoxib to patients with BE for 10 days significantly decreased COX-2 expression, PGE_2_ contents and PCNA of epithelium of BE [44]. Furthermore, the Chemoprevention for Barrett's Esophagus Trial (CBET) has started in 2003 as a phase IIb, multicenter, randomized, double-masked, placebo-controlled study of CLX in patients with Barrett's dysplasia [45]. Unfortunately, the CBET failed to prove to prevent progression of Barrett's dysplasia to cancer [46]. But, the CBET study group measured the surface area affected by BE using quantitative endoscopy at baseline and at one year after treatment and compared the CLX group and the placebo group [47]. It demonstrated that the area of Barrett's involvement in the CLX group showed larger reduction than in the placebo group. Thus it remains undecided whether COXIBs are able to prevent the development of EAC. The AspECT described above will be expected to show positive results [28].

#### 5.2.4. Other Agents

Ursodeoxycholic acid (UDCA) and gugglesterone are other candidates for chemoprevention of esophageal carcinogenesis. UDCA does not stimulate COX-2 or Cdx2 mRNA expression so much as DCA in the experiment using Barrett's epithelial cancer cells [48]. UDCA is clinically used for some inflammatory diseases such as primary biliary cirrhosis, primary sclerosing cholangitis, and ulcerative colitis. Guggulsterone, a plant sterol, has anti-inflammatory and antitumour effects, functioning as an antagonist for the farnesoid X receptor. It suppresses bile acid-induced Cdx 2 expression in gut-derived adenocarcinoma cells [49]. But, the influences of both agents on inflammation-related adenocarcinogenesis in esophagus remain elucidated.

## 6. Conclusions

The inflammation-metaplasia-adenocarcinoma sequence in the development of EAC is one of representatives of inflammation-related carcinogenesis model in gastrointestinal cancer. Since the incidences of BE and EAC are rapidly increasing, effective prevention should be established as soon as possible. Chemoprevention is an ideal method because of less invasiveness compared to endoscopic surveillance. Most promising agents are NSAIDs and PPIs. In this sense the AspECT will have important meanings to use these drugs for prevention of EAC.

## Figures and Tables

**Table 1.** Preventive effects on esophageal adenocarcinogenesis of COXIBs and NSAIDs using rodent reflux models.

**Reporter (year)**	**Oyama (2005)**	**Buttar (2002)**		**Chen (2002)**
Rat strain	Fisher 344	Sprague-Dawlay		Sprague-Dawlay
Reflux model	Schlatter $	Levrat #		EGDA ++

Chemical agent	Celecoxib	MF-tricyclic	Sulindac	Sulindac
Initiation of agent (week)	1	4	4	4
Sacrifice (week)	40	28	28	40

Incidence of lesion			
Barrett's esophagus			
Control	89%	80%	80%	---
Treated	25%	83%	60%	---
	(p < 0.005)	(not significant)	(not significant)	
Adenocarcinoma			
Control	47%	51%	51%	58%
Treated	0%	23%	11%	27%
	(p < 0.05)	(p = 0.013)	(p < 0.001)	(p < 0.05)

$ refer to Figure 2B;

# refer to Figure 1B;

++ refer to Figure 1C

**Table 2.** Incidence of regenerative changes, Barrett's esophagus and adenocarcinoma.

**Weeks**	**Group**	**No. of animals**	**Incidence (%) of**							

			**RT**		**BCH**		**BE**		**Carcinoma**	
10	Control	10	100	*	100	*	10		0	
	Celecoxib	5	40		40		0		0	
20	Control	10	100	*	100	*	40		0	
	Celecoxib	5	40		40		20		0	
30	Control	10	100	*	100	*	50		10	
	Celecoxib	5	40		40		40		0	
40	Control	19	100	*	100	*	89	**	47	*
	Celecoxib	8	38		38		25		0	

RT: regenerative thickening; BCH: basal cell hyperplasia; BE: Barrett's esophagus

** p < 0.005;

* p < 0.05, Fisher's extract test

## References

[1] Pera M. (2000). Epidemiology of esophageal cancer, especially adenocarcinoma of the esophagus and esophagogastric junction. Recent Results Cancer Res..

[2] Miwa K., Segawa M., Takano Y., Matsumoto H., Sahara H., Yagi M., Miyazaki I., Hattori T. (1994). Induction of oesophageal and forestomach carcinomas in rats by reflux of duodenal contents. Br. J. Cancer.

[3] Miwa K., Sahara H., Segawa M., Kinami S., Sato T., Miyazaki I., Hattori T. (1996). Reflux of duodenal or gastro-duodenal contents induces esophageal carcinoma in rats. Int. J. Cancer.

[4] Pera M., Cardesa A., Bombi J.A., Ernst H., Pera C., Mohr U. (1989). Influence of esophagojejunostomy on the induction of adenocarcinoma of the distal esophagus in Sprague-Dawley rats by subcutaneous injection of 2,6-dimethylnitrosomorpholine. Cancer Res..

[5] Attwood S.E., Smyrk T.C., DeMeester T.R., Mirvish S.S., Stein H.J., Hinder R.A. (1992). Duodenoesophageal reflux and the development of esophageal adenocarcinoma in rats. Surgery.

[6] Fein M., Peters J.H., Chandrasoma P., Ireland A.P., Oberg S., Ritter M.P., Bremner C.G., Hagen J.A., DeMeester T.R. (1998). Duodenoesophageal reflux induces esophageal adenocarcinoma without exogenous carcinogen. J. Gastrointest. Surg..

[7] Goldstein S.R., Yang G.Y., Curtis S.K., Reuhl K.R., Liu B.C., Mirvish S.S., Newmark H.L., Yang C.S. (1997). Development of esophageal metaplasia and adenocarcinoma in a rat surgical model without the use of a carcinogen. Carcinogenesis.

[8] Ireland A.P., Peters J.H., Smyrk T.C., DeMeester T.R., Clark G.W., Mirvish S.S., Adrian T.E. (1996). Gastric juice protects against the development of esophageal adenocarcinoma in the rat. Ann. Surg..

[9] Miyashita T., Ohta T., Fujimura T., Ninomiya I., Fushida S., Hattori T., Miwa K. (2006). Duodenal juice stimulates oesophageal stem cells to induce Barrett's oesophagus and oesophageal adenocarcinoma in rats. Oncol. Rep..

[10] Mukaisho K., Miwa K., Kumagai H., Bamba M., Sugihara H., Hattori T. (2003). Gastric carcinogenesis by duodenal reflux through gut regenerative cell lineage. Dig. Dis. Sci..

[11] Eberhard D., Tosh D. (2007). Transdifferentiation and metaplasia as a paradigm for understanding development and disease. Cell Mol. Life Sci..

[12] Tatsuta T., Mukaisho K., Sugihara H., Miwa K., Tani T., Hattori T. (2005). Expression of Cdx2 in early GRCL of Barrett's esophagus induced in rats by duodenal reflux. Dig. Dis. Sci..

[13] Kazumori H., Ishihara S., Rumi M.A., Kadowaki Y., Kinoshita Y. (2006). Bile acids directly augment caudal related homeobox gene Cdx2 expression in oesophageal keratinocytes in Barrett's epithelium. Gut.

[14] Marchetti M., Caliot E., Pringault E. (2003). Chronic acid exposure leads to activation of the cdx2 intestinal homeobox gene in a long-term culture of mouse esophageal keratinocytes. J. Cell Sci..

[15] Souza R.F., Krishnan K., Spechler S.J. (2008). Acid, bile, and CDX: The ABCs of making Barrett's metaplasia. Am. J. Physiol. Gastrointest. Liver Physiol..

[16] Sato F., Meltzer S.J. (2006). CpG island hypermethylation in progression of esophageal and gastric cancer. Cancer.

[17] Nishijima K., Miwa K., Miyashita T., Kinami S., Ninomiya I., Fushida S., Fujimura T., Hattori T. (2004). Impact of the biliary diversion procedure on carcinogenesis in Barrett's esophagus surgically induced by duodenoesophageal reflux in rats. Ann. Surg..

[18] Hofstetter W.L., Peters J.H., DeMeester T.R., Hagen J.A., DeMeester S.R., Crookes P.F., Tsai P., Banki F., Bremner C.G. (2001). Long-term outcome of antireflux surgery in patients with Barrett's esophagus. Ann. Surg..

[19] Csendes A. (2004). Surgical treatment of Barrett's esophagus: 1980-2003. World J. Surg..

[20] Csendes A., Bragheto I., Burdiles P., Smok G., Henriquez A., Parada F. (2006). Regression of intestinal metaplasia to cardiac or fundic mucosa in patients with Barrett's esophagus submitted to vagotomy, partial gastrectomy and duodenal diversion. A prospective study of 78 patients with more than 5 years of follow up. Surgery.

[21] Sporn M.B., Dunlop N.M., Newton D.L., Smith J.M. (1976). Prevention of chemical carcinogenesis by vitamin A and its synthetic analogs (retinoids). Fed. Proc..

[22] Abrams J.A. (2008). Chemoprevention of esophageal adenocarcinoma. Ther. Adv. Gastroenterol..

[23] Miyashita T., Shah F.A., Marti G.P., Wang J., Bonde P., Gibson M.K., Ohta T., Montgomery E.A., Duncan M., Harmon J.W. (2011). Rabeprazole impedes the development of reflux-induced esophageal cancer in a surgical rat model. Dig. Dis. Sci..

[24] Hillman L.C., Chiragakis L., Shadbolt B., Kaye G.L., Clarke A.C. (2004). Proton-pump inhibitor therapy and the development of dysplasia in patients with Barrett's oesophagus. Med. J. Aust..

[25] El-Serag H.B., Aguirre T.V., Davis S., Kuebeler M., Bhattacharyya A., Sampliner R.E. (2004). Proton pump inhibitors are associated with reduced incidence of dysplasia in Barrett's esophagus. Am. J. Gastroenterol..

[26] García Rodríguez L.A., Lagergren J., Lindblad M. (2006). Gastric acid suppression and risk of oesophageal and gastric adenocarcinoma: A nested case control study in the UK. Gut.

[27] Farrow D.C., Vaughan T.L., Sweeney C., Gammon M.D., Chow W.H., Risch H.A., Stanford J.L., Hansten P.D., Mayne S.T., Schoenberg J.B., Rotterdam H., Ahsan H., West A.B., Dubrow R., Fraumeni J.F., Blot W.J. (2000). Gastroesophageal reflux disease, use of H2 receptor antagonists, and risk of esophageal and gastric cancer. Cancer Causes Control.

[28] Das D., Chilton A.P., Jankowski J.A. (2009). Chemoprevention of oesophageal cancer and the AspECT trial. Recent Results Cancer Res..

[29] Sasaki S., Miwa K., Fujimura T., Oba M., Miyashita T., Kinami S. (2007). Ingestion of thioproline suppresses rat esophageal adenocarcinogenesis caused by duodenogastroesophageal reflux. Oncol. Rep..

[30] Kumagai H., Mukaisho K., Sugihara H., Miwa K., Yamamoto G., Hattori T. (2004). Thioproline inhibits development of esophageal adenocarcinoma induced by gastroduodenal reflux in rats. Carcinogenesis.

[31] Iijima K., Grant J., McElroy K., Fyfe V., Preston T., McColl K.E. (2003). Novel mechanism of nitrosative stress from dietary nitrate with relevance to gastro-oesophageal junction cancers. Carcinogenesis.

[32] Farrow D.C., Vaughan T.L., Hansten P.D., Stanford J.L., Risch H.A., Gammon M.D., Chow W.H., Dubrow R., Ahsan H., Mayne S.T., Schoenberg J.B., West A.B., Rotterdam H., Fraumeni J.F., Blot W.J. (1998). Use of aspirin and other nonsteroidal anti-inflammatory drugs and risk of esophageal and gastric cancer. Cancer Epidemiol. Biomark. Prev..

[33] Wilson K.T., Fu S., Ramanujam K.S., Meltzer S.J. (1998). Increased expression of inducible nitric oxide synthase and cyclooxygenase-2 in Barrett's esophagus and associated adenocarcinomas. Cancer Res..

[34] Zimmermann K.C., Sarbia M., Weber A.A., Borchard F., Gabbert H.E., Schror K. (1999). Cyclooxygenase-2 expression in human esophageal carcinoma. Cancer Res..

[35] Morris C.D., Armstrong G.R., Bigley G., Green H., Attwood S.E. (2001). Cyclooxygenase-2 expression in the Barrett's metaplasia-dysplasia-adenocarcinoma sequence. Am. J. Gastroenterol..

[36] Kauer W.K., Peters J.H., DeMeester T.R., Ireland A.P., Bremner C.G., Hagen J.A. (1995). Mixed reflux of gastric and duodenal juice is more harmful to the esophagus than gastric juice alone. The need for surgical therapy re-emphasized. Ann. Surg..

[37] Stein H.J., Barlow A.P., DeMeester T.R., Hinder R.A. (1992). Complications of gastroesophageal reflux disease: Role of the lower esophageal sphincter, esophageal acid acid/alkaline exposure and duodenogastric reflux. Ann. Surg..

[38] Zhang F., Altorki N.K., Wu Y.C., Soslow R.A., Subbaramaiah K., Dannenberg A.J. (2001). Duodenal reflux induces cyclooxygenase-2 in the esophageal mucosa of rats: evidence for involvement of bile acids. Gastroenterology.

[39] Song S., Guha S., Liu K., Buttar N.S., Bresalier R. (2007). COX-2 induction by unconjugated bile acids involves reactive oxygen species-mediated signaling pathways in Barrett's oesophagus and oesophageal adenocarcinoma. Gut.

[40] Chen X., Li N., Wang S., Hong J., Fang M., Yousselfson J., Yang P., Newman R.A., Lubet R.A., Yang C.S. (2002). Aberrant arachidonic acid metabolism in esophageal adenocarcinogenesis, and the effects of sulindac, nordihydroguaiaretic acid, and alpha-difluoromethylornithine on tumorigenesis in a rat surgical model. Carcinogenesis.

[41] Buttar N.S., Kenneth K.W., Leontovich O., Westcott J.Y., Pacifico R.J., Anderson M.A., Krishnadath K.K., Lutzke L.S., Burgart L.J. (2002). Chemoprevention of esophageal adenocarcinoma by COX-2 inhibitors in an animal model of Barrett's esophagus. Gastroenterology.

[42] Oyama K., Fujimura T., Ninomiya I., Miyashita T., Kinami S., Fushida S., Ohta T., Miwa K. (2005). A COX-2 inhibitor prevents the esophageal inflammation-metaplasia-adenocarcinoma sequence in rats. Carcinogenesis.

[43] Futagami S., Suzuki K., Hiratsuka T., Shindo T., Hamamoto T., Tatsuguchi A., Ueki N., Shinji Y., Kusunoki M., Wada K., Miyake K., Gudis K., Tsukui T., Sakamoto C. (2006). Digestion. Celecoxib inhibits Cdx2 expression and prevents gastric cancer in Helicobacter pylori-infected Mongolian gerbils. Digestion.

[44] Kaur B.S., Khamnehei N., Iravani M., Namburu S.S., Lin O., Tradafilopoulos G. (2002). Rofecoxib inhibits cyclooxygenase 2 expression and activity and reduce cell proliferation in Barrett's esophagus. Gastroenterology.

[45] Heath E.I., Canto M.I., Wu T.T., Piantadosi S., Hawk E., Unalp A., Gordon G., Forastiere A.A. (2003). CBET Research Group. Chemoprevention for Barrett's esophagus trial. Design and outcome measures. Dis. Esophagus..

[46] Heath E.I., Canto M.I., Piantadosi S., Montgomery E., Weinstein W.M., Herman J.G., Dannenberg A.J., Yang V.W., Shar A.O., Hawk E., Forastiere A.A. (2007). Chemoprevention for Barrett's Esophagus Trial Research Group. Secondary chemoprevention of Barrett's esophagus with celecoxib: Results of a randomized trial. J. Natl. Cancer Inst..

[47] Shar A.O., Gaudard M.A., Heath E.I., Forastiere A.A., Yang V.W., Sontag S.J. (2009). Modeling using baseline characteristics in a small multicenter clinical trial for Barrett's esophagus. Contemp. Clin. Trials.

[48] Burnat G., Majka J., Konturek P.C. (2010). Bile acids are multifunctional modulators of the Barrett's carcinogenesis. J. Physiol. Pharmacol..

[49] Yamada T., Osawa S., Hamaya Y., Furuta T., Hishida A., Kajimura M., Ikuma M. (2010). Guggulsterone suppresses bile acid-induced and constitutive caudal-related homeobox 2 expression in gut-derived adenocarcinoma cells. Anticancer Res..

